# *FOXC2* Autoregulates Its Expression in the Pulmonary Endothelium After Endotoxin Stimulation in a Histone Acetylation-Dependent Manner

**DOI:** 10.3389/fcell.2021.657662

**Published:** 2021-05-04

**Authors:** Sheng Xia, Wei Yu, Heather Menden, Scott T. Younger, Venkatesh Sampath

**Affiliations:** ^1^Department of Pediatrics, Children’s Mercy Kansas City, MO, United States; ^2^Center for Pediatric Genomic Medicine, Children’s Mercy Kansas City, MO, United States

**Keywords:** FOXC2, autoregulation, endothelium, sepsis, lung

## Abstract

The innate immune response of pulmonary endothelial cells (EC) to lipopolysaccharide (LPS) induces Forkhead box protein C2 (FOXC2) activation through Toll Like Receptor 4 (TLR4). The mechanisms by which FOXC*2* expression is regulated in lung EC under LPS stimulation remain unclear. We postulated that FOXC2 regulates its own expression in sepsis, and its transcriptional autoregulation directs lymphatic EC cell-fate decision. Bioinformatic analysis identified potential FOXC2 binding sites in the *FOXC2* promoter. In human lung EC, we verified using chromatin immunoprecipitation (ChIP) and luciferase assays that FOXC2 bound to its own promoter and stimulated its expression after LPS stimulation. Chemical inhibition of histone acetylation by garcinol repressed LPS-induced histone acetylation in the *FOXC2* promoter region, and disrupted LPS-mediated FOXC2 binding and transcriptional activation. CRISPR/dCas9/gRNA directed against FOXC2-binding-element (FBE) suppressed LPS-stimulated FOXC2 binding and autoregulation by blocking FBEs in the *FOXC2* promoter, and repressed expression of lymphatic EC markers. In a neonatal mouse model of sterile sepsis, LPS-induced FOXC2 binding to FBE and FOXC2 expression in lung EC was attenuated with garcinol treatment. These data reveal a new mechanism of LPS-induced histone acetylation-dependent *FOXC2* autoregulation.

## Introduction

FOXC2 is a member of the forkhead box (FOX) transcription factor family (Seo et al., 2006). It plays a critical role in vascular development. FOXC2 is a key transcription regulator involved in VEGF regulated vascular formation and remodeling in physiological and pathological conditions. VEGFA activates FOXC2 through kinase insert domain receptor (KDR) pathway in arterial endothelium, and then FOXC2 binds to delta like canonical Notch ligand 4 (DLL4) promoter and upregulates DLL4 expression, which in turn activates Notch signaling (Hayashi and Kume, 2008). Subsequently hes related family bHLH transcription factor with YRPW motif 1 (HEY1) and HEY2, downstream of DLL4/Notch signaling, collaborating with SRY-box transcription factor 7 (Sox7)/Sox18, lead to arterial specification through EphrinB2 activation (Hayashi and Kume, 2008). Unrelated to the role of FOXC2 in developmental signaling, we showed that systemic lipopolysaccharide (LPS) upregulates *Dll4* expression through TLR4-ERK-FOXC2 axis to program endothelial cell (EC) specification and induce inflammatory angiogenesis in the neonatal mouse lung (Xia et al., 2018).

Loss of one copy of *FOXC2* in humans causes hereditary lymphedema distichiasis (LD) syndrome and primary valve failure in veins of lower extremities (Finegold et al., 2001). *Foxc2* haploinsufficient mice serve as a model for human LD syndrome (Fang et al., 2000), while *Foxc2* homozygous conventional knockout mice die embryologically after day E13.5 up to shortly after birth because of branchial arch and skeletal anomalies (Kume, 2009). In lymphatic endothelial cells, VEGFC/VEGFR3 activates FOXC2 to regulate lymphatic development, growth, function and survival (Petrova et al., 2004). FOXC2 also plays an important role in the later stages of lymphatic development by regulating the morphogenesis of lymphatic valves, stabilize postnatal lymphatic vasculature, and interactions of the lymphatic endothelium with vascular mural cells (Sabine et al., 2015). In pathological states, FOXC2 overexpression is involved in cancer progression through several mechanisms, including epithelial mesenchymal transition (EMT) (Mani et al., 2007; Cui et al., 2015; Paranjape et al., 2016). FOXC2-AS1 overexpression promotes proliferation and migration of vascular smooth muscle cells and tumor cells (Yang et al., 2019; Zhang et al., 2019). Regulation of FOXC2 expression in native and diseased states is not fully understood. The miR-548c-5p clusters regulates FOXC2 transcription (Christofides et al., 2019) and LncRNA FOXC2-AS1 stabilizes *FOXC2* mRNA by forming double stranded RNA to promote FOXC2 expression (Zhang et al., 2017). Histone deacetylase 5 (HDAC5) negatively regulates FOXC2 expression during mouse embryonic development (Lagha et al., 2010). The methylation and histone acetylation of chromatin structure plays essential roles in regulation of EC function (Ohtani and Dimmeler, 2011; Zhou et al., 2011; Li et al., 2020). HDAC5 represses angiogenic genes in EC, and VEGFA induces HDAC5 nuclear export, which allows histone acetyltransferases (HATs) to acetylate transcriptional factors and histones to activate gene expression (Urbich et al., 2009; Zhang et al., 2013; Fish et al., 2017). As a key transcriptional regulator in VEGF signaling pathways (Hayashi and Kume, 2009), FOXC2 binding affinity to Fox-binding element (FBE) may be regulated by chromatin acetylation in EC.

Bronchopulmonary dysplasia (BPD) is a chronic lung disease that develops in premature babies exposed to hyperoxia and sepsis. The aberrant vasculature and alveolar structure were observed in the lungs in which BPD developed (Thébaud and Abman, 2007; Baker and Abman, 2015). Srinivasan et al. (2017) found that over-represented SNPs in proximity to *FOXC2* in premature infants with all sepsis, which indicates *FOXC2* expression level decides susceptibility to infection in neonatal lungs. Our previous study shows that *FOXC2* expression is stimulated by LPS in the mouse lung endothelium and in isolated human pulmonary microvascular endothelial cells (HPMEC) (Xia et al., 2018), but how LPS stimulates *FOXC2* expression remains unknown. Considering the varied roles played by FOXC2 in embryonic development and pathological states, it is imperative to examine the mechanisms underlying *FOXC2* expression. In this study, we hypothesized that FOXC2 expression is self-regulated by FOXC2 transcriptional activation and its binding affinity to FBE is regulated by histone acetylation during sepsis.

## Materials and Methods

### Cell Culture and Reagents

Immortalized HPMEC (HPMEC-Im) was generated as described before (Nitkin et al., 2020). Briefly, primary human lung EC purchased from a commercial source (ScienCell) underwent lentiviral transformation to generate an immortal cell line, and immunostaining for PECAM1, ERG and oxLDL uptake were used to confirm EC specificity. Primary HPMEC and HPMEC-Im were grown in endothelial cell medium (ECM) supplemented with fetal bovine serum (FBS), antibiotics, and endothelial cell growth serum (ECGS) as recommended by the manufacturer (ScienCell) in a humidified incubator containing 5% CO_2_ at 37°C. HPMEC-Im were transfected overnight with the indicated plasmids or empty plasmids (mock) with Lipofectamine 3000 (Thermo-Fisher) as per the manufacturer’s protocol. Ultrapure LPS was purchased commercially from Invivogen. Garcinol was purchased from Santa Cruse. For experiments with inhibitors, primary HPMEC and HPMEC-Im were pre-treated with 25 μM Garcinol for 45 min prior to the addition of LPS (0.5 μg/ml).

### Animal Model

Care of mice before and during the experimental procedures was conducted in accordance with the policies at the University of Missouri- Kansas City Lab Animal Resource Center (Protocol 1510) and the National Institutes of Health guidelines for the care and use of laboratory animals. All protocols had prior approval from the University of Missouri- Kansas City Institutional Animal Care and Use Committee. Wildtype C57BL/6 strain was obtained commercially from Charles River (Burlington, MA). LPS injections (2 mg/kg) to the mice were given intraperitoneally (i.p), and sterile saline used for controls (Sigma, St. Louis, MO) with or without 25 μmol/kg Garcinol pretreatment. Mice were then euthanized using a 100 μl i.p injection pentobarbital, exsanguinated with the cessation of a heartbeat, and the lungs were harvested and utilized as described below.

### Plasmids and Lentiviral Vector

The FOXC2 CA, generated by cloning the FOXC2 DBD in-frame with the VP16 transcriptional activation domain (Gerin et al., 2009), was a gift from Dr. Ormond MacDougald. pIRES-Puro-EGFP-FOXC2 CA was generated as described before (Xia et al., 2018). Human *FOXC2* gene was amplified with 5′-tgagctagccca ccatgcaggcgcgctactccgtgtccga-3′ and 5′-agtctcgagtcagtatttcgtgc agtcgtaggagtaggg-3′ from HPMEC genomic DNA and cloned into pIRES-Puro-EGFP (Addgene) to generate pIRES-Puro-EGFP-FOXC2 WT. Oligos 5′-caccgtccgggattcctagaggga-3′ and 5′-aaactccctctaggaatcccggac-3′; 5′-caccgcgagggaaactcagtttgt-3′ and 5′-aaacacaaactgagtttccctcgc-3′; 5′-caccgattggctcaaagttccggg-3′ and 5′-aaaccccggaactttgagccaatc-3′ were annealed to generate double-strand gRNA templates next to FBEs about −1.7, −0.9, and −0.45 kb in *FOXC2* promoter and then cloned into phU6-gRNA plasmid, respectively, to generate phU6-gRNA targeting FBEs in FOXC2 promoter for FBE dCas9/gRNA assay. 0.45 and 2 kb upstream DNA of *FOXC2* promoter regions were amplified with 5′-actgctagccgctttcagcaagaagacttttgaaacttttcc-3′ and 5′-tgagagcgagagagcgcgagaga-3′; 5′-tattggaaataagtggcacgcc-3′ and 5′-tgagagcgagagagcgcgagaga-3′ from HPMEC genomic DNA and cloned into pGL4.10 to generate pGL4.10-FOXC2-Pomoter respectively for luciferase assay.

Lentiviral vector containing the dCas9 (Gilbert et al., 2013; Richardson et al., 2018) and packing vectors, psPAX2 and pMD2.G, were purchased from Addgene. These plasmids were transfected into HEK293T (Takara) using calcium phosphate transfection to produce lentivirus containing dCas9. After 2 days of transfection, supernatant containing lentiviral particles were harvest and used to transduce HPMEC-Im cells in the presence of polybrene (Santa Cruz) at 8 μg/ml.

### Luciferase Assay

pGL4.10 -FOXC2-Promoter, pGL4.75 Renilla and pIRES-Puro-EGFP-FOXC2 WT (or CA) plasmids were co-transfected into HPMEC-Im cultured in 96-well plate. Next day, luciferase assay was applied with Dual-Glo^®^ Luciferase Assay System (Promega) according to the manufacturer’s instructions.

### Chromatin Immunoprecipitation (ChIP)

HPMEC-Im were treated with LPS for 0.5 h, with or without garcinol pretreatment, and then ChIP was applied according to the manufacturer’s instructions (Thermo Fisher Scientific). Rabbit anti FOXC2 antibody (Abcam, Cambridge, MA) was used to pull down FOXC2 binding DNA and rabbit anti acetylated H3K27antibody (Abcam, Cambridge, MA) was used to pull down H3K27ac binding DNA. qPCR was performed to quantify pulled-down DNA with *FOXC2* promoter primers: −1.7 kb, 5′-cccgtgtttagccttgttaaag-3′ and 5′-ctaggaatcccggacagtttg-3′; −0.9 kb, 5′-cctcgataggttatccttgacg-3′ and 5′-tggattggaatggcaggg-3′; −0.5 kb, 5′-tgattggctcaaagttccgg-3′ and 5′-aagaggccaagtcccttttag-3′.

Mice were treated with LPS for 3 h, with or without garcinol pretreatment, and then lung EC were isolated. ChIP was applied according to the manufacturer’s instructions (Thermo Fisher Scientific, Rockford, IL). Anti-FOXC2 antibody (Abcam, Cambridge, MA) was used to pull down FOXC2 binding DNA. qPCR was performed to quantify pulled-down DNA with mouse *Foxc2* promoter primers: 5′-cgactggagatgttgaaggaa-3′ and 5′-attttatgccaaccttgacgc-3′

### CRISPR/dCas9/gRNA

HPMEC-Im was transduced by lentivirus containing dCas9 and then screened with 200 mg/L hygromycin to generate dCas9 stable cell line HPMEC-Im/dCas9. Mixed FBE gRNAs were transfected into dCas9 stable HPMEC.

### Isolation of Murine Endothelial Cells

For endothelial cell isolation, all lobes of the lung from 2 neonatal C57BL/6 pups (4–7 days old) were pooled per condition. The isolation of mouse lung endothelial cells was done as described previously (Menden et al., 2019).

### Quantification of mRNA Expression Using Quantitative RT-PCR (qRT-PCR)

Total RNA was extracted from HPMEC and mouse lung EC using the PureLink RNA Mini Kit (Life Technologies) following the manufacturer’s instruction and cDNA was synthesized from 1 μg of RNA using an iScript cDNA synthesis kit (Bio-Rad, Hercules, CA) according to the manufacturer’s instruction. qRT-PCR was run on a Bio-Rad IQ5 with SYBR green master mix (Bio-Rad). The primers for mouse and human target genes, and mouse *Actb* and human 18S were purchased commercially from Sigma. mouse *Actb* and human 18S were used as the housekeeping gene. The relative gene expression was calculated using the Pfaffl method.

### Immunoprecipitation for Phosphorylation Studies

HPMEC-Im grown to the 90% confluence in 60-mm dishes had various treatments, and lysates were used for immunoprecipitation studies. It was done as described previously (Xia et al., 2018).

### Immunoblotting for Quantifying Changes in Protein Expression

HPMEC-Im and mouse lung tissue were homogenized in RIPA lysis buffer containing commercially available protease and phosphatase inhibitors (Sigma) with after LPS treatment, with the clarified lysates used for immunoblotting. Immunoblotting was done following standard protocol. The primary antibodies used were: goat anti-FOXC2 [Santa Cruz Biotechnology (SCBT), Santa Cruz, CA], mouse anti- CRISPR-Cas9 (Abcam, Cambridge, MA), mouse anti-phospho-Serine [(p)Ser], mouse anti-phospho-Threonine [(p)Thr] and mouse anti-ACTB (Sigma). Densitometry was performed using ImageJ Software (NIH, Bethesda, MD) and changes were normalized to ACTB.

### Immunofluorescent (IF) Staining

IF was done as in our previous study (Menden et al., 2019). The lungs of the mouse pups were fixed in formalin and frozen, and sections were cut onto slides. The slides were stained with rabbit anti FOXC2 antibody (23066-1-AP, Thermo Fisher Scientific, Rockford, IL) and rat anti PECAM antibody (550274, BD Biosciences, San Jose, CA). *n* = 3–4 per group.

### Statistical Analysis

Data are presented as mean ± SD or median with interquartile range. *P* < 0.05 was considered significant. For cell culture experiments, data are from a minimum of three independent experiments with adequate technical replicates used for quantification. All animal data were obtained in littermate controls. For animal experiments, a minimum of 3 animals were used for each experimental group. RNA quantification and PCR results had 2–3 technical replicates. For all data, we initially examined whether distribution of data was Gaussian using the D’Agostino-Pearson omnibus normality test. If data were normally distributed, then ANOVA with a *post hoc* Tukey test was used for analysis. If data did not meet Gaussian assumptions, a Mann-Whitney *U*-test was used for analysis. For most analysis, fold-changes were calculated related to expression/changes in untreated controls. Statistical analysis was done using Graphpad Prism 7.0 (San Diego, CA).

### Study Approvals

Lab experiments were reviewed and approved under the University of Missouri-Kansas City IBC, protocol number 18–28. Animal experiments were reviewed and approved under the University of Missouri-Kansas City IACUC, protocol number 1510-02.

## Results

### FOXC2 Expression Is Upregulated by LPS and FOXC2-CA in HMPEC-Im

**FIGURE 1:**
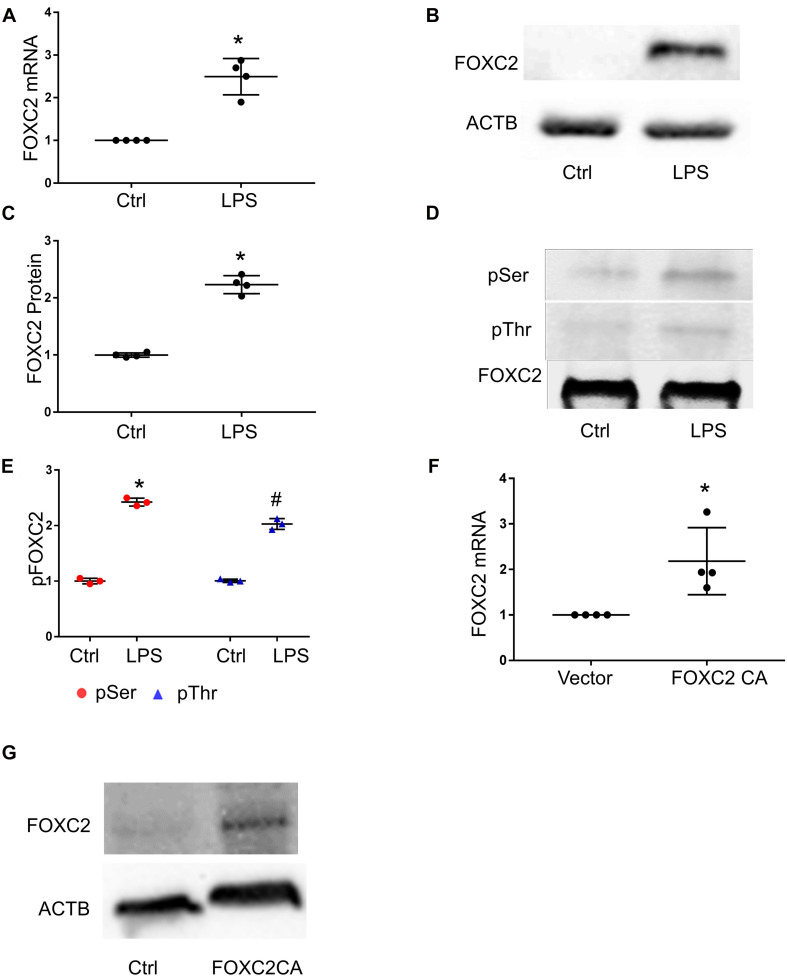
FOXC2 expression is upregulated by LPS and FOXC2 CA in HMPEC-Im. **(A)** *FOXC2* mRNA was assessed by qRT-PCR 4 h after 0.5 μg/ml LPS treatment. *n* = 4 per group; *p < 0.01, LPS vs. Ctrl. **(B)** FOXC2 protein was quantified by immunoblotting after overnight 0.5 μg/ml LPS treatment, with densitometry shown graphically **(C)**. *n* = 4 per group; *p < 0.05, LPS vs. Ctrl. **(D)** FOXC2 protein was immunoprecipitated from cell lysates at 30 min after LPS, and serine and threonine phosphorylation was assessed by immunoblotting, with densitometry shown graphically **(E)**. *n* = 3 per group, *p < 0.0001, LPS pSer vs. Ctrl pSer; ^#^*p* < 0.005, LPS pThr vs. Ctrl pThr. **(F)** Cells were collected 2 days after transfection of *FOXC2C CA* plasmid and empty control plasmid respectively, and *FOXC2* mRNA was quantified. *n* = 4 per group; *p < 0.05, FOXC2 CA vs. vector. **(G)** Immunoblotting showed FOXC2CA stimulated FOXC2 expression.

To study whether LPS stimulates *FOXC2* expression in HPMEC-Im, we compared *FOXC2* mRNA and protein expression with or without LPS stimulation. qRT-PCR showed *FOXC2* mRNA expression increased 6 h after LPS treatment (Figure 1A), and immunoblotting demonstrated protein expression increased 18 h after LPS treatment (Figures 1B,C). Serine and threonine phosphorylation of FOXC2, required for its transcriptional activation (Ivanov et al., 2013), was induced by LPS in HPMEC-Im at 30 min (Figures 1D,E). To determine whether FOXC2 can stimulate its own expression, FOXC2-CA, a peptide containing the nuclear binding domain of FOXC2, was transfected and overexpressed in HPMEC-Im. We noted that *FOXC2* mRNA expression increased (Figure 1F) using qRT-PCR with primers that specifically detect endogenous *FOXC2* mRNA. FOXC2 protein expression was also increased after FOXC2-CA transfection (Figure 1G). These data reveal that LPS activates FOXC2 protein by serine and threonine phosphorylation and stimulates FOXC2 expression potentially through a positive feedback loop.

### Luciferase Assay Demonstrates That FOXC2 Promoter Is Activated by FOXC2 WT and FOXC2 CA

**FIGURE 2:**
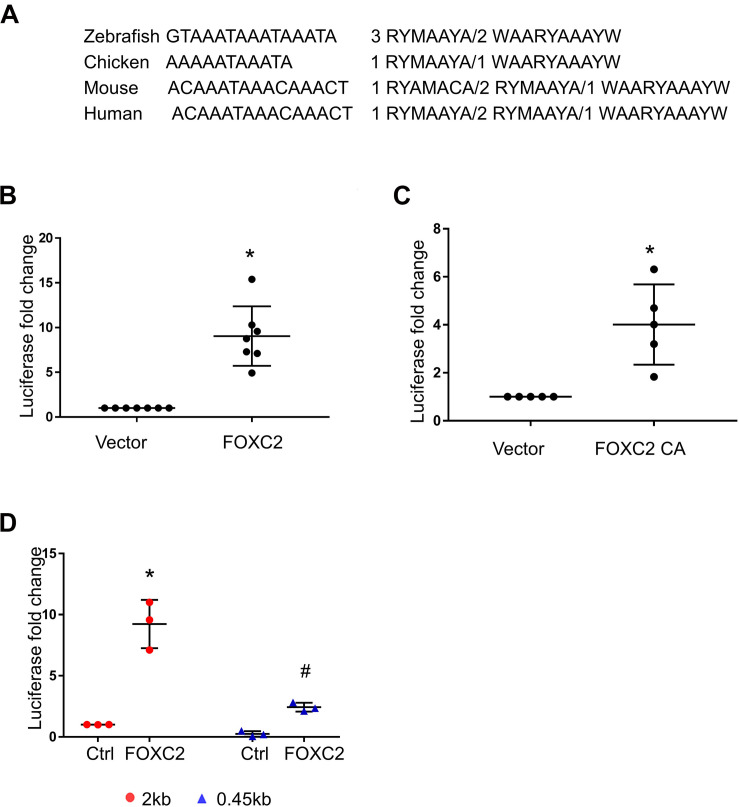
Luciferase assay demonstrates that FOXC2 promoter is activated by FOXC2 WT and FOXC2 CA in HPMEC-Im. **(A)** Overlapped FBEs in Zebrafish, Chicken, mouse and human in 2 kb FOXC2 upstream region. **(B,C)** 2 kb DNA upstream sequence of human *FOXC2* transcriptional start site (TSS) was cloned into pGL4.10 (luc2) and co-transfected with expression plasmids containing *FOXC2 CA* and *FOXC2 WT*, respectively. **(B)** FOXC2 WT overexpression stimulated luciferase reporter activity. *n* = 7 per group; *p < 0.001. **(C)** FOXC2 CA increased luciferase activity. *n* = 5 per group, *p < 0.005. **(D)** 0.45 kb upstream sequence of *FOXC2 TSS* was cloned into pGL4.10 (luc2) and co-transfected with *FOXC2 WT* plasmid. FOXC2-induced luciferase activity of 0.45 kb promoter was 3.8-fold less than that of the 2 kb promoter. *n* = 3 per group; *p < 0.05, FOXC2-stimulated 2 kb vs. 2 kb; ^#^*p* < 0.01, FOXC2-stimulated 2 kb vs. FOXC2-stimulated 0.45 kb.

As we found that FOXC2 CA can stimulate *FOXC2* expression in HPMEC-Im, we studied whether FOXC2 directly regulates self-expression in HPMEC-Im. Analyzing the *FOXC2* promoter sequences in several species on PROMO server, we found potential FBEs in the *FOXC2* promoter regions of zebra fish, chicken, mouse and human (Figure 2A). So, we posited that FOXC2 binds to and regulates its own promoter. 17 potential FBEs predicted by JASPAR, whose relative scores are more than 0.8, distribute from −2000 to −456 bp upstream of human *FOXC2* gene (Table 1). We next cloned the 2 kb human *FOXC2* upstream DNA sequence into a luciferase reporter plasmid. Luciferase assays revealed that the promoter activity increased 9- and 4-fold with FOXC2 WT and CA overexpression, respectively (Figures 2B,C). We then truncated the 2 kb *FOXC2* upstream DNA to 0.45 kb containing no FBE, placed it in front of luciferase reporter and performed luciferase assays. We found that 0.45 kb activity was much lower than 2 kb activity when FOXC2 WT was over expressed (Figure 2D). These data indicate that FOXC2 may activate its own transcriptional activity through FBEs.

**TABLE 1:** 17 potential FEBs predicted by JASPAR, whose relative scores are more than 0.8, distribute from −456 to −2000 bp upstream of human *FOXC2* mRNA (NM_005251.2).

**Sequence**	**Score**	**Relative score**	**Strand**
CAAATAAACAAA	12.813	0.933425979	+
GGAATAAATAAT	7.92393	0.856966601	+
TAAATAAGTATA	7.78859	0.854849912	+
TCCACAAATAAA	6.97316	0.84209754	+
AAAATAAATTTT	6.88391	0.840701689	+
TAATCAAATAAT	6.83889	0.839997682	−
TAAATAATCAGT	6.60866	0.836397124	+
AAAGTAAAAACT	6.14274	0.82911063	+
TCAGTCCACAAA	6.13537	0.828995446	+
AAAAAAATCAAT	5.84931	0.824521662	−
CAACAAAACAAA	5.73341	0.822709108	+
TGTCTCAACATC	5.58709	0.820420851	+
AAAACAAAAAAA	5.45669	0.818381568	+
TGATTAAATAAG	5.44639	0.818220387	+
CAAATAATTAAT	5.15087	0.813598808	−
AATACAAATGTT	4.68596	0.806328132	+
CACCTCAATAAT	4.30528	0.800374752	−

### LPS Enhances FOXC2 Expression by Stimulating Histone Acetylation and FOXC2 Binding Affinity

**FIGURE 3:**
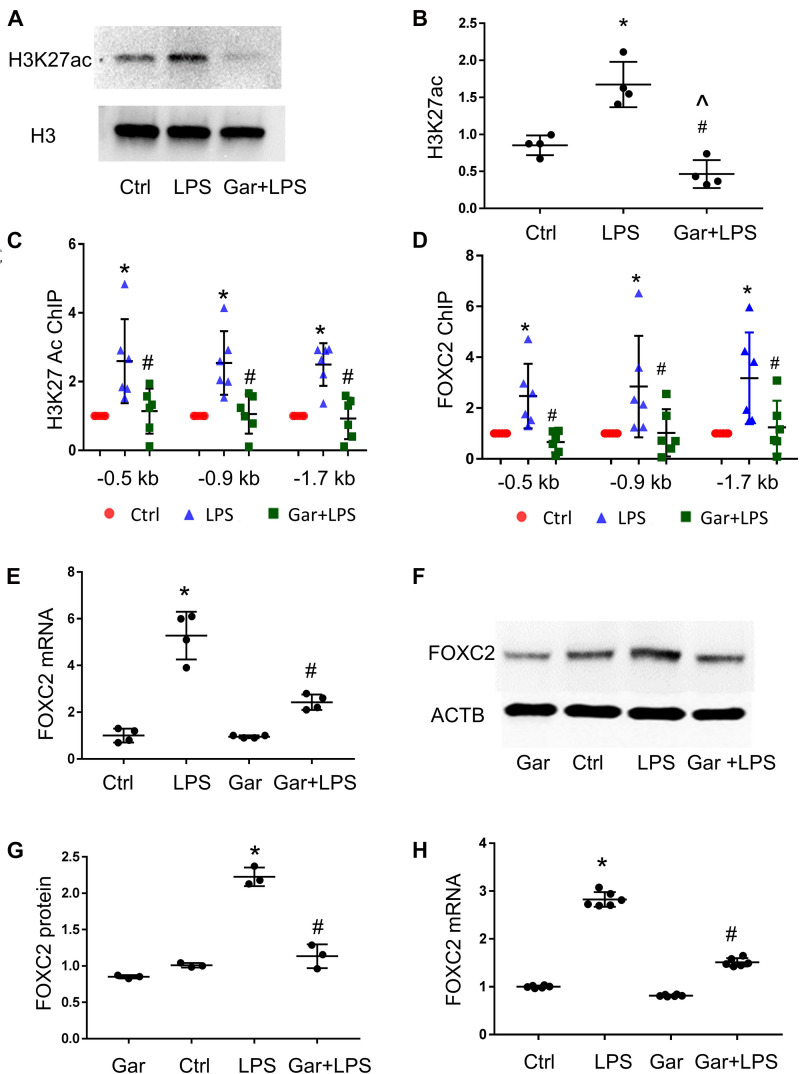
LPS enhances FOXC2 expression by stimulating histone acetylation and FOXC2 binding affinity in HPMEC. **(A)** Acetylated H3K27 (H3K27ac) protein was quantified by immunoblotting from HPMEC-Im cell lysates 30 min after LPS with or without Garcinol (Gar) pretreatment, with densitometry shown graphically **(B)**. *n* = 4 per group; *p < 0.05, LPS vs. Ctrl; ^#^*p* < 0.05, LPS + Gar vs. LPS, ^*p* < 0.05, LPS + Gar vs. Ctrl. **(C)** ChIP with anti-acetylated H3K27 antibody was performed at 30 min after LPS treated HPMEC-Im, with or without garcinol pretreatment. *n* = 6 per group; *P < 0.01, LPS vs. ctrl; ^#^*p* < 0.05, LPS + Gar vs. LPS. **(D)** ChIP with anti-FOXC2 antibody was performed 30 min after LPS treated HPMEC-Im, with or without garcinol pretreatment. *n* = 6 per group; *P < 0.05, LPS vs. ctrl; ^#^*p* < 0.05, LPS + Gar vs. LPS. **(E)** *FOXC2* mRNA was quantified by qRT-PCR 4 h after LPS treated HMPEC-Im, with or without garcinol pretreatment. *n* = 4 per group; *P < 0.01, LPS vs. ctrl; ^#^*P* < 0.05, LPS + Gar vs. LPS. **(F)** FOXC2 protein was quantified by immunoblotting after 16 h LPS treated HPMEC-Im, with or without garcinol pretreatment, with densitometry shown graphically **(G)**. *n* = 3 per group; *p < 0.005, LPS vs. ctrl; ^#^*p* < 0.001, LPS + Gar vs. LPS. **(H)** *FOXC2* mRNA was quantified by qRT-PCR 4 h after LPS treated primary HPMEC, with or without garcinol pretreatment. *n* = 6 per group; *P < 0.0001, LPS vs. ctrl; ^#^*P* < 0.0001, LPS + Gar vs. LPS.

Luciferase assay demonstrated that over-expressed FOXC2 activates its own promoter, so we next determined to identify the mechanisms by which LPS induces *FOXC2* expression. Histone lysine acetylation and deacetylation is essential chromatin modification for genes epigenetic regulation (Grunstein, 1997; Jenuwein and Allis, 2001). The expression of several genes in the VEGF pathway is regulated by HDACs (Fraineau et al., 2015) and *FOXC2* expression is regulated by HDAC5 in mouse embryos (Lagha et al., 2010), so we posited that LPS induces FOXC2 expression by stimulating histone acetylation within the FOXC2 promoter region. To test this hypothesis, three pairs of primers were designed to cover two FBE enriched locations at −1.7 and −0.9 kb, and the FBE from –445 to −456 bp (Supplementary Figure 1). At first, we checked histone acetylation in response to LPS. Immunoblotting showed that H3K27 was widely acetylated 30 min after LPS treatment (Figures 3A,B), consistent with previous reports (Lauterbach et al., 2019). To investigate histone acetylation in FOXC2 promoter region, we performed ChIP assay with anti-H3K27ac antibody and confirmed that three locations in *FOXC2* promoter were acetylated in response to 30-min LPS treatment (Figure 3C). P300/CBP is a major histone acetyl-transferase that is stimulated by LPS (Hassa et al., 2003; Liu et al., 2018), so we used garcinol, an inhibitor specifically targeting p300/CBP(IC50 approximately 7 μM) and P300/CBP-associated factor complex(IC50 approximately 5 μM) (Balasubramanyam et al., 2004), repressed LPS-induced acetylation of FOXC2 promoter sites queried (Figure 3C). To study whether histone acetylation in FBE influences FOXC2 binding, we applied ChIP with anti-FOXC2 antibody and revealed that FOXC2 binding to those three locations was increased with LPS treatment, but it was significantly suppressed with garcinol pretreatment (Figure 3D).

To study whether histone acetylation and FOXC2 binding affinity in FOXC2 promoter region regulates FOXC2 expression in HPMEC-Im, we did qRT-PCR and immunoblotting. We demonstrated that FOXC2 LPS-stimulated FOXC2 mRNA and protein expression levels were suppressed with garcinol pretreatment (Figures 3E–G). We also confirmed that LPS stimulated *FOXC2* expression is suppressed with garcinol pretreatment in primary HPMEC (Figure 3H). These studies identify that histone acetylation in FOXC2 promoter region is important to LPS-stimulated FOXC2 binding and *FOXC2* expression.

### CRISPR/dCas9/FBE gRNA Blocks FOXC2 Binding and Represses Autoregulation

**FIGURE 4:**
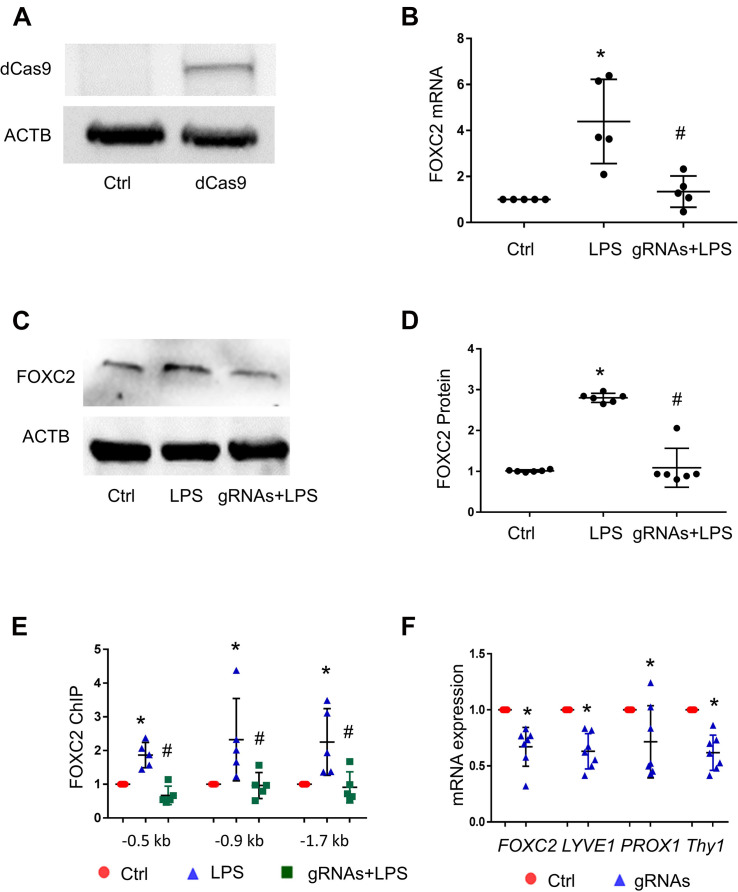
CRISPR/dCas9/FBE gRNA blocks LPS-stimulated FOXC2 binding, represses autoregulation in HPMEC-Im, and alters EC character. **(A)** dCas9 protein expression in stable cell line of HPMEC-Im/dCas9 was shown by immunoblotting, but no expression in control HPMEC-Im. **(B)** *FOXC2* RNA expression was quantified by qTR-PCR 2 days after gRNA mix were transfected into HPMEC-Im/dCas9, with or without 4-h LPS treatment. *n* = 5 per group, *p < 0.05, LPS vs. Ctrl; ^#^*p* < 0.05, gRNAs + LPS vs. LPS. **(C)** FOXC2 protein was quantified by immunoblotting from cell lysate 6 h after LPS treatment with or without gRNAs transfection, with densitometry shown graphically **(D)**. *n* = 6, *p < 0.01, LPS vs. ctrl; ^#^*P* < 0.001, gRNAs + LPS vs. LPS. **(E)** ChIP with anti-FOXC2 antibody was performed 30 min after LPS treatment of gRNAs transfected HPMEC-Im/dCas9. *n* = 5 per group, *p < 0.05, LPS vs. ctrl; ^#^*p* < 0.05 gRNAs + LPS vs. LPS. **(F)** FOXC2, LYVE-1, PROX-1 and Thy1 RNA expression was quantified 2 days after gRNAs were transfected into HPMEC-Im/dCas9. *n* = 7 per group, *p < 0.05.

To confirm that FOXC2 binding to its promoter upregulates its expression in pathological conditions, we utilized CRISPR/dCas9, a nuclease dead Cas9 retaining the ability to bind to target DNA based on the gRNA targeting sequence, to investigate whether blocking FBE can prevent FOXC2 binding and inducing transcriptional activity. At first, we generated dCas9 stable HPMEC-Im cell line with lentivirus and confirmed the cells expressed dCas9 with immunoblotting (Figure 4A). Three gRNAs mixture targeting FBE enriched regions in *FOXC2* promoter, was transfected into dCas9stable expressed HPMEC-Im. LPS-induced *FOXC2* mRNA and protein expression was reduced dramatically after 4-h and overnight LPS treatments, respectively in FBE gRNAs transfected cells by comparing to scramble gRNA transfected cells (Figures 4B–D). To verify whether reduced *FOXC2* mRNA expression was caused by FBE blocking, we applied ChIP and found that LPS-induced FOXC2 binding to FBEs was totally inhibited by FBE gRNA/dCas9 (Figure 4E). These data verify that FBEs in FOXC2 promoter plays an important role of regulating *FOXC2* expression in response to LPS, FBE blocking by gRNA/dCas9 prevents FOXC2 binding and autoregulation.

### FBE Blocking in *FOXC2* Promoter Suppresses Expression of Lymphatic EC Markers

FOXC2 regulates lymphatic development and programs lymphatic EC specification (Petrova et al., 2004; Sabine et al., 2015). To investigate whether repression of *FOXC2* expression through blocking FBEs alters EC characters in HPMEC-Im, we tested lymphatic EC markers 2 days after gRNAs transfection and revealed that lymphatic EC markers (Podgrabinska et al., 2002; Wilting et al., 2002; Shinoda et al., 2016), such as *PROX1*, *LYVE1*, and *THY1*, were reduced with repression of FOXC2 expression (Figure 4F). These data suggest that FOXC2 autoregulation may play a role in lymphatic EC specification.

### FOXC2 Autoregulation in Mouse Lung EC

**FIGURE 5:**
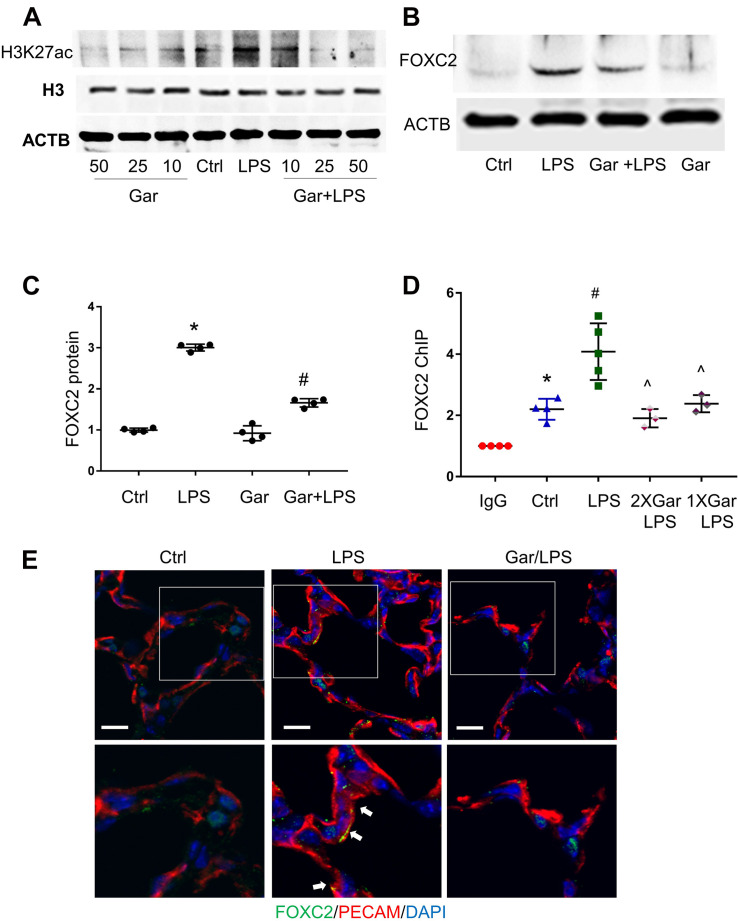
LPS-induced FOXC2 autoregulation is histone acetylation dependent in mouse lung. **(A)** Mouse lungs were collected after 3 h LPS treatment, with or without different dosages of garcinol pretreatment (2 h). Protein expression of histone H3 and acetylated histone H3 was shown by immunoblotting. **(B)** Mouse lung EC were isolated after 3 h LPS treatment at P5, with or without 2 h garcinol pretreatment. FOXC2 protein in mouse lung EC was quantified by immunoblotting, with densitometry shown graphically **(C)**. *n* = 4 per group, *p < 0.0001, LPS vs. Ctrl; ^#^*p* < 0.0001, Gar + LPS vs. LPS. **(D)** Mouse lung EC were isolated after 3 h LPS with or without garcinol co-treatment) at P7. ChIP with anti-FOXC2 antibody was performed with mouse lung EC. *n* ≥ 3 per group; *p < 0.05, ctrl vs. IgG; ^#^*p* < 0.05, LPS vs. ctrl; ^*p* < 0.05, LPS + Gar vs. LPS. **(E)** Mouse lungs were cryopreserved at P7 after 24 h LPS treatment, with or without garcinol pre-treatment. FOXC2 (green) and PECAM (red) IF staining was applied. Lower panels present the magnified images in the boxes of the up panels. White arrows point to the EC (red) which express FOXC2 (green). *n* = 3–4 per group; scale bars: 10 μm.

To confirm our *in vitro* finding *in vivo*, we evaluated LPS-induced histone acetylation, FOXC2 binding and expression in the developing mouse lung. Day of life 7 mouse pups were treated with intraperitoneal LPS, with or without garcinol pretreatment. At first, we treated with mice with different dosage of garcinol and found that 25 μmol/kg garcinol efficiently blocked LPS-induced H3K27 acetylation (Figure 5A). We next examined the effect of garcinol on LPS-induced FOXC2 expression in P7 mouse lung EC. Immunoblotting lung EC lysates (PECAM pull down) showed that LPS-induced FOXC2 expression was repressed by 25 μmol/kg garcinol (Figures 5B,C). Additionally, immunofluorescence studies indicated that FOXC2 lung EC expression (PECAM – red; FOXC2-green) induced by LPS at 24 h was inhibited with garcinol (Figure 5E). There is only one FBE-enriched location in mice (Figure 2A), and ChIP with anti-FOXC2 antibody revealed that LPS enhanced FOXC2 binding to this location, and this binding was repressed by garcinol pretreatment in P7 mouse lung EC (Figure 5D). These data revealed that LPS stimulated FOXC2 expression through the same mechanism in HPMEC *in vitro* and mouse lung EC *in vivo*.

## Discussion

FOXC2 is a major transcriptional regulator of lymphatic development and EC phenotype specification (Petrova et al., 2004; Sabine et al., 2015; Xia et al., 2018). FOXC2 phosphorylation regulates FOXC2-mediated transcription in lymphatic EC and pulmonary EC (Ivanov et al., 2013; Xia et al., 2018). Previous studies reveal that Foxc2 expression is regulated by miR-548c-5p clusters, LncRNA FOXC2-AS1 and HDAC5 (Lagha et al., 2010; Zhang et al., 2017; Christofides et al., 2019). While our previous study showed that LPS strongly induces FOXC2 in lung and retinal EC *in vivo* and *in vitro* the mechanisms were not determined (Xia et al., 2018). In this study we demonstrate that FOXC2 autoregulates its own expression, under native and inflammatory states. Using gain of function and loss of function approaches we identify that FBEs in the FOXC2 promoter are the key of self-regulation. We demonstrate FOXC2 binding affinity to its promoter is enhanced by histone acetylation in response to LPS, and repression of histone acetylation inhibits its autoregulatory expression in human and mouse lung EC. Interestingly, blocking FBEs using a CRISPR/dCas9/gRNA strategy strongly represses FOXC2 autoregulation, and suppresses the expression of markers typically associated with lymphatic EC specification. Our data uncover a novel mechanism of *FOXC2* self-regulation in EC, potentially significant to FOXC2’s developmental and pathological role.

Prior work demonstrated that FOXC2 expression is regulated by LPS in primary HPMEC (Xia et al., 2018). Primary HPMEC are fragile, and so we generated HPMEC-Im because it facilitates stable gene-modification such as dCas9 through several passages unlike primary EC. We overexpressed FOXC2 CA and confirmed that FOXC2 stimulated its mRNA and protein expression in HMPEC-Im. Subsequent studies where a 2 kb DNA upstream promoter region of FOXC2 gene was used to regulate luciferase expression revealed that both FOXC2 WT and CA upregulated its promoter’s transcription activity. However, FOXC2 overexpression and luciferase assay can’t distinguish whether FOXC2 regulates its transcription expression directly or indirectly. We analyzed *FOXC2* promoter regions in zebrafish, chicken, mouse and human, and found putative FBE in all of them. So, we hypothesized that FOXC2 could bind to its promoter and regulate its transcription expression.

JASPAR (jaspar.genereg.net) predicted 17 potential FBEs (Table 1), whose relative scores are more than 0.8 and which distribute from −2000 to −456 bp upstream of human *FOXC2* gene (Supplementary Figure 1). Three overlapped FBE hotspots were found: 6 putative FBEs localize between −1752 to −1782, 2 putative sites are between −1572 to −1588, and 3 putative sites are between −915 to −940. We also searched FBE in 2 kb DNA fragment manually. According to FOXC binding motif as “RYAMACA” (R = G/A, Y = T/C, M = A/C) (Chen et al., 2019), there is only one potential FBE, CAAATAAACAAA, which represents the highest JASPAR relative score and localizes between −1752 to −1782. However, according to FBE as “RYMAAYA” and “WAARYAAAYW” (Kaufmann and Knöchel, 1996; Carlsson and Mahlapuu, 2002; Samatar et al., 2002; Hayashi and Kume, 2008), three putative FBEs locate between −1752 to −1782 as JASPAR predicted, and two lie at –942 and −632 separately, which are not predicted by JASPAR. With collective consideration, we deigned two pairs of primers for ChIP and two gRNAs for dCas9 repressor, which target two putative FBE hotspots, −1752 to −1782 and −915 to −940 separately, and one pair of primers target the first FBE, which locates at −456. Luciferase assay demonstrated that 0.45 kb *FOXC2* upstream DNA significantly lost self-regulation function, indicating those FBEs are necessary for FOXC2 autoregulation. Retained FOXC2-induced activity of 0.45 kb may be cause by FOXC2 indirect binding or regulation. To uncover that, more studies will be conducted.

Most genes in VEGF signaling pathway are regulated by HDACs and HATs (Urbich et al., 2009; Zhang et al., 2013; Fish et al., 2017), which change chromatin accessibility and regulate gene transcription (Voss and Thomas, 2018). LPS is known to induce histone modification (Hamon and Cossart, 2008). Loss of HDAC5 function stimulates *Foxc2* expression in mouse embryos (Lagha et al., 2010). So, we posited that *FOXC2* autoregulated self-expression might be dependent on histone acetylation after stimulation by LPS. We demonstrate by ChIP that FOXC2 binds to its promoter and its binding affinity is enhanced by LPS treatment. Our data also reveal that garcinol, a HAT inhibitor, that is specific to p300 and PCAF (Balasubramanyam et al., 2004; Kim et al., 2020; Wang et al., 2020; Kopytko et al., 2021), represses LPS-stimulated histone acetylation at *FOXC2* promoter, FOXC2 binding affinity and FOXC2 expression in HPMEC-Im. Our data shows that LPS-stimulated FOXC2 expression in primary HPMEC is also repressed by garcinol consistent with our data in immortalized HPMEC. Our data, while consistent with other studies showing that LPS promotes histone acetylation uncover FOXC2 as a target for histone acetylation dependent FOXC2 expression. Although our studies clearly demonstrate the role of histone acetylation in FOXC2 expression both *in vivo* and *in vitro*, a minor limitation is that we did not identify the precise histone acetylase that mediates FOXC2 promoter acetylation.

To validate FOXC2 binds its promoter, we used a loss of function approach by blocking two putative FBE hotspots (−1752 to −1782 and −915 to −940) and one FBE at −456 in the upstream of FOXC2 gene utilizing CRISPR/dCas9/gRNAs technologies. We identify that dCas9/gRNAs specifically target those FBEs and repress LPS-stimulated *FOXC2* expression with ChIP, qRT-PCR and immunoblotting, respectively. After we revealed the mechanism of *FOXC2* autoregulation in human lung EC *in vitro*, we pursued studies to confirm FOXC2 autoregulation *in vivo*. With the same strategy we predicted FBEs in mouse *Foxc2* upstream DNA, we only found one FBE hotspot (Figure 2A) within 2 kb mouse *Foxc2* upstream DNA. With ChIP, we demonstrated that FOXC2 bound to the FBEs hotspot in mouse lung EC after intraperitoneal LPS injections, and garcinol pretreatment repressed FOXC2 binding and LPS-induced FOXC2 expression *in vivo*. These data uncover that LPS-induced FOXC2-mediated autoregulation is HAT dependent in mouse lung EC. FOXC1 and FOXC2 are closely related FOX family members and collaboratively work together to regulates VEGF downstream genes in arterial EC (Seo et al., 2006). Both ChIP and dCas9/gRNA can’t differentiate FOXC2 binding to its promoter directly or indirectly, and EMSA can’t define it too when two FOXC member proteins, which share the same FBE, are added. So, in this study, we only applied ChIP and gRNA/dCas9 to prove that FOXC2 binds to its promoter and regulates its expression.

To study how *FOXC2* autoregulation influences EC cell fate, we transfected mixed FBE-targeted gRNAs into dCas9 stable expressed HPMEC-Im and analyzed expression of lymphatic markers. After blocking FOXC2 autoregulation, EC had decreased expression of lymphatic EC specification markers, which suggests that *FOXC2* autoregulation may play an important role in EC programing and differentiation during lung development. CRISPR/dCas9 strategy is broadly used to study DNA-protein interaction and gene expression regulation, but dCas9 footprint covers 78.1 bp ± 37.9 bp (Josephs et al., 2015). So, the other transcription factor binding element may be blocked when dCas9/gRNAs target to FBEs. To overcome this disadvantage, mutations of FBEs in *FOXC2* promoter will be studied in the future.

Collectively, using human lung EC, as well as a sterile sepsis model in neonatal mice, we demonstrate that FOXC2 regulates its own expression through a histone acetylation- dependent positive feedback loop during TLR4 stimulation in developing lung EC. Our data demonstrates a direct link between lung EC innate immune signaling and FOXC2 self-regulation as suggested by our previous study (Xia et al., 2018). Further, we show that suppression of FBE on the FOXC2 promoter using CRISPR/dCas9/gRNA strategy suppresses the native expression of lymphatic EC specification markers such as *Lyve1* and Thy1. The implications of FOXC2 transcriptional activation and autoregulation during LPS stimulation to lung EC inflammatory signaling and fate specification in chronic models of inflammation is an area of future investigation. Further, whether exaggerated FOXC2 autoregulation underlies FOXC2’s role in cancer remains to be determined.

## Data Availability Statement

The original contributions presented in the study are included in the article/Supplementary Material, further inquiries can be directed to the corresponding author/s.

## Ethics Statement

The animal study was reviewed and approved by Care of mice before and during the experimental procedures was conducted in accordance with the policies at the University of Missouri- Kansas City Lab Animal Resource Center (Protocol 1510) and the National Institutes of Health guidelines for the care and use of laboratory animals. All protocols had prior approval from the University of Missouri- Kansas City Institutional Animal Care and Use Committee.

## Author Contributions

VS and SX: conception and design. SX, WY, and HM: data collection. VS, SX, WY, HM, and SY: analysis and interpretation, drafting and editing the manuscript. All authors contributed to the article and approved the submitted version.

## Conflict of Interest

The authors declare that the research was conducted in the absence of any commercial or financial relationships that could be construed as a potential conflict of interest.
