# Supplementary material for: FOXC2 Autoregulates Its Expression in the Pulmonary Endothelium After Endotoxin Stimulation in a Histone Acetylation-Dependent Manner
Source: Front Cell Dev Biol. 2021 May 4;9:657662. doi: 10.3389/fcell.2021.657662 (PMC8129010; doi:10.3389/fcell.2021.657662)
Supplement: Supplementary file 1 [file Data_Sheet_1.DOCX]

T**TGTCTCAACATC**CTTTTGCTGAGAATCGAATACGCAGCCGATGAACAGCCAGGAAGGGTGCAAGGAAACCTGA**AATACAAATGTT**CTCCCTGAAGCCCTCTTCCCTGCCCAACCAGACCAGCAACTTCCAAAATTCTGCCCGTGTTTAGCCTTGTTAAAGGGGTGTCTCACTCCTTCAGGGAAAGTGGGAAAAGGGGATCTG**ATTATTGAGGTG**TGGAA**GGAA*TA*AATAATCAGTCCACAAA*TAAACAAA****CT*GTCCGGGATTCCTAGAGGGAAGGAGAAATCCTTGAAGGAGATCCAAGTCGCTCCAGGTCTGCCTGCCGAATAATATCATCCCGAAGGGATCTTGAACCGTTTGCAATCAACCGCTCACCCAGTCTTCCCACGGAGCGCGCTCCCTAACTCACCCTACCCACC**CAACAAAACAAAAAAA**AGGCTGAAATATAGAAAAGCAACTTGGAGGCTCCCAGGGGGACGTTGCCAGGAGCAGGAGGCAGGGACAGCGCCCTAGGGTCGGTGTTAGCGGCCGGCGCCGGCCTGGGCCACGGGAAACGTCCACGCTTGGTGCCCGCGGTGCGCGGCGCTCATTGCGCGCGCCTTCGAGCCAAGCCCCCGCGGAAAACAGGCTCGGGTTTCTCCTCGCAGGGCCCAGGAACTCGGCTCTGCCTGGCCCGGGTGGGTCGCTGCATTGTCCCGGTCTTCTGGGAGTGCGGGGTCAGCTTGTTAGAGGGAATTTCTACCTGGGAAAAGGGAGACGAGTTTCGAAGCTGAAGTTGGTAGGCTGCGAGTGTCCACGCGGGAGACGAAAGGGGGAAATAGCAGAGTCACTTCACCCTTTTCCCCAAACCCCACAAAACTGCTCGCAGCGACGCGGATGATCTACCGAATTCCCCGCGAATTCGGAGGATTAAGTTGTCAGTCAGCACGTTGCTACCTTCCCCTCTATGCACTCCGCTGCCTGGCTCCTCGGCGGGGAGCGAGGGAAACTCAGTTTGTAGGGTTTACCTCTAAAACCTCGATAGGTTATCCTTGACGACCCCGAGCCTGGAAACTCCCTGTTGATG**ATTAATTATTTGATTAAATAAGTATA**ACATCCAGGAGAGGCCCTGCCATTCCAATCCAGCGCGTTTGCTTTGAATCCATTACACCTGGGCCCCCATAATTAGGAAATCTAATTATTCGCTTCATCACTCATTAATAAGAAAAATGTCCCAGGATCATTGCTACTTACAAGGTCTTTGGGAGAGATATTTTACTCTATTAATCCATTCTATTTTATATTTCAA**ATTGATTTTTTT**TAACAGAGGAAAGTGGCTATCTTTTTGTTTTGGGCATGTGGGCCCATTCACCAAAATGTGATCAT***AAAATAAATT*TT**AATAAGATATAACTTTTTAAAAAGTTTTCAAGTGAAGACGGAGTCGCCGCGGAGGCCGGGGCGGCGGGGTCTTAGAGCCGACGGATTCCTGCGCTCCTCGCCCCGATTGGCGCCGACTCCTCTCAGCTGCCGGGTGATTGGCTCAAAGTTCCGGGAGGGGGCGTGGCCCGAGG**AAAGTAAAAACT**CGCTTTCAGCAAGAAGACTTTTGAAACTTTTCCCAATCCCTAAAAGGGACTTGGCCTCTTTTTCTGGGCTCAGCGGGGCAGCCGCTCGGACCCCGGCGCGCTGACCCTCGGGGCTGCCGATTCGCTGGGGGCTTGGAGAGCCTCCTGCGCCCCTCCTCGCGCGGGCCGAGGGTCCACCTGGGTCCCCAGGCCGCGGCGTCTCCGCTGGGTCCGCGGCCGCCCGCCTGCCCGCGCTGCCGCCGCCGGGTCCTGGAGCCAGCGAGGAGCGGGGCCGGCGCTGCGCTTGCCCGGGGCGCGCCCTCCAGGATGCCGATCCGCCCGGTCCGCTGAAAGCGCGCGCCCCTGCTCGGCCCGAGCGCCGCCGCCCGCGCACCCTCGCCCCGGAGGCTGCCAGGAGCCCGGGGCCGCCCCTCCCGCTCCCCTCCTCTCCCCCTCTGGCTCTCTCGCGCTCTCTCGCTCTCAGGGCCCCCCTCGCTCCCCCGGCCGCAGTCCGTGCGCGAGGGCGCCGGCGAGCCGTCTCGGAAGCAGCATGCAGGCGC

**Supplemental Figure 1.** **Human *FOXC2* upstream DNA sequence with 5’ FOXC2 mRNA sequence:** *FOXC2* 5’ mRNA (NM_005251.2) is marked by underline. FEBs predicted by JASPAR is marked as **bold**; RYAMACA is marked as red; WAARYAAAYW is marked as *italic*; RYMAAYA is turquoise highlighted. The primers for ChIP are yellow highlighted with line arrows.
